# Vascular response to the microcirculation in the fingertip by local vibration with varied amplitude

**DOI:** 10.3389/fbioe.2023.1197772

**Published:** 2023-06-12

**Authors:** Lizhong Mu, Aoran Sun, Youqiang Chen, Huimin Chen, Jianda Li, Bingqi Linghu, Hang Zhou, Qingzhuo Chi, Xiaofeng Luan, Yue Pan

**Affiliations:** ^1^ Key Laboratory of Marine Energy Utilization and Energy Conservation, School of Energy and Power, Dalian University of Technology, Dalian, China; ^2^ The Combination of Medicine and Engineering of Cardiovascular Fluid Dynamics Key Laboratory of Liaoning Province, Shenyang, China; ^3^ The Second Hospital Affiliated Dalian Medical University, Dalian, China; ^4^ Department of General Surgery, Central Hopspital of Dalian University of Technology, Dalian, China; ^5^ College of Biomedical Engineering, Dalian University of Technology, Dalian, China

**Keywords:** hand-transmitted vibration, amplitude, LDF, fingertip blood flow, silicone-endothelial cell model, inflammatory factor, wavelet analysis

## Abstract

**Objectives:** We investigated the effect of local vibration intensity on the vascular response to the microcirculation of the finger.

**Materials and methods:** We performed hand-transmitted vibration experiments combined with laser Doppler flowmetry (LDF) to measure the blood perfusion signals of fingertips in the vibrated hand and the contralateral middle finger under the same frequency and different amplitude vibration, and to analyze the changes of microcirculatory blood perfusion levels in the fingers, and to investigate the effects of vibration stimulation on the endothelial, neural and myogenic regulatory frequency ranges of fingertips based on wavelet analysis. Furthermore, the transparent silicone films were fabricated and cultured with vascular endothelial cell (EC), which will undergo the local vibration with varied amplitude. And the expression of inflammatory factors was detected in the ECs.

**Results:** Low-frequency vibration leads to a decreased blood flow in fingertip, and the degree of reduction in fingertip blood flow increases as the amplitude gradually increases, and the period required for blood flow to return to normal level after hand-transmitted vibration gradually increases. The decrease in blood flow is more pronounced in the vibrating hand than in the contralateral hand. In addition, nuclear factor-κB (NF-κB) expression increased significantly with the increase of vibration amplitude.

**Conclusion:** High amplitude vibrations caused the inflammatory reaction of ECs which will lead to the altered endothelial regulatory activity. The endothelial regulatory activity is closely related to the blood perfusion in the microcirculation.

## 1 Introduction

According to recent statistics, hand-arm vibration disease has become a common occupational disease, mostly seen in workers who use hand-held vibrating tools or come into contact with vibrating workpieces in production. It is mainly due to periodic mechanical vibrations or shocks directly acting on or transmitted to the practitioner’s arm, causing impairment of the peripheral circulation and nerve function of the hand, which in turn leads to damage to the microcirculation of the upper limb (Wu and Yang, 2019), seriously affecting the quality of life of the patient.

Currently, the pathogenesis of hand-arm vibration disease is not clear, but it is closely related to damage to blood vessels and nerves caused by periodic vibration, for example. The common factors that induce hand-arm vibration disease are: vibration intensity, frequency, and duration of vibration (Chen and Lin, 2008). Long-term exposure of the hand to vibration stimuli will induce peripheral microcirculatory and neurological dysfunction in the hand, which will then develop into vibratory microangiopathy.

It is clinically important to investigate the effect of vibration stimulation on the peripheral blood microcirculation of the hand. On the one hand, long-term low-frequency and high-amplitude vibration can lead to the development of vibration disease. This is manifested by the disruption of nerve reflexes and neurohumoral system. This is due to the fact that low-frequency vibration interferes with the function of skin receptors, nerves and nerve trunks, resulting in increased secretion of norepinephrine by synaptic nerve endings. The inability of synaptic nerve endings to capture norepinephrine properly leads to its massive entry into the bloodstream, causing increased vascular tension, which in turn induces vasospasm. (Stoyneva et al., 2003) emphasized that static loads and vibrations will affect the dorsal microcirculatory function of the hand, which is closely related to the development of Raynaud’s syndrome. Mechanical vibrations force an increase in contact pressure at the fingertip in contact with the finger, causing severe deformation of the arterial blood vessel wall and changes in blood flow to the fingertip (Wu et al., 2002). (Ye and Griffin, 2016) found that when a vibration of 125 Hz was applied to the index finger, blood flow was reduced to a greater extent in both directly vibrated and unvibrated fingers. Therefore, it was inferred that there was a sympathetic response in the dorsal microcirculation of the hand under local vibration stimulation (Griffin et al., 2006). Results of three-dimensional finite element analysis of the mechanical response of the fingertip to dynamic loading showed that the resonance frequency of the fingertip was approximately 100 Hz. the soft tissue of the fingertip would have a higher local stress and strain response under dynamic loading. The soft tissues of the nail bed are less affected by vibration compared to the ventral region of the finger, which means that changes in vascular structure and function are likely to occur in the areas that are deformed the most after being subjected to vibration (Wu et al., 2008).

On the other hand, high-frequency low-amplitude vibration has a positive effect on tissue microcirculation. Not only does it stimulate tissue metabolism, but it also promotes the restoration of blood microcirculatory system function (Cardinale and Wakeling, 2005). It has been shown by that vibration stimulation of specific intensity contributes to the healing of diabetic wounds (Yu et al., 2017). (Ichioka et al., 2011) found that vibration stimulation improves blood microcirculatory function, as well as NO plays an important role in the vasodilatation process induced by vibration stimulation. In addition, (Nakagami et al., 2007) found that vibration response played a positive role in the treatment of ulcers through animal experiments. Animal experiments have shown that shock waves can improve microcirculation and have a therapeutic effect on limb ischemia (De Sanctis et al., 2000). (Malysheva and Petrov, 2015) investigated the effect of vibratory stimulation on microhemodynamics during the development of vibropathy. Currently, the use of biometric methods can assist in the detection of peripheral polyneuropathy (NemtsovSmirnova and Timina, 2000; Bregovsky et al., 2015) such as diabetic distal polyneuropathy and rheumatoid arthritis, and these applications suggest the relevance of vibration to the physiology and pathology of the peripheral circulatory system.

In addition, wavelet analysis methods can be used for the analysis and processing of physiological signals and are more widely used. (Cheng, 2020) extracted the fluctuation components of skin temperature signals in the endothelial frequency band by wavelet analysis to compare the differences between healthy patients and diabetic patients before, during, and after heating. (Zhu et al., 2020) used wavelet analysis to investigate the changes in the amplitude of blood flow fluctuations before and after vibration at different vibration frequencies by performing local vibration on the foot.

In addition, local vibratory stimuli tend to cause the development of inflammatory response in endothelial cells (ECs), and inflammatory factors are one of the key factors involved in the inflammatory response (Yang et al., 2022). Nuclear factor κB (NF-κB) is one of the more important inflammatory factors and plays an important role in the gene regulation of the inflammatory response (Dobrovolskaia and Kozlov, 2005). It has been shown that abnormal expression of NF-κB is associated with the development of various diseases, such as diabetes, cardiovascular diseases, and cancer, *etc.* The interconnection between NF-κB and NO is also close, and excessive expression of NF-κB will directly contribute to the abnormal secretion function of NO (Janssen-Heininger et al., 2000), leading to changes in vasodilatory capacity and causing certain risks to human health.

Therefore, clarifying the mechanism of action of vibration stimulation on peripheral circulation blood flow promotion or inhibition, effectively reducing harmful vibration stimulation and increasing beneficial vibration stimulation to human body will have important clinical value for the prevention of vibration disease. In this study, hand-transmitted vibration experiments were conducted to measure fingertip skin blood flow values under different vibration amplitudes using a vibration device and a blood flow collection device. Blood perfusion level analysis and wavelet analysis were used to investigate the effects of different amplitudes of local vibration on fingertip blood flow. Examining the effects of vibration stimulation on the endothelial, neural and myogenic regulatory frequency bands of the fingertip plays an important role in revealing the mechanism of action of vibration stimulation on peripheral circulating blood flow.

## 2 Materials and methods

### 2.1 Overview of vibration experiment subjects and experimental environment

Eight healthy male volunteers, aged 21–25 years, with a height range of 170–180 cm and a weight range of 70–80 kg, all had no experience of frequent use of hand-held vibrating tools, no cardiovascular or neurological disorders, and no common family history of genetic disorders, were included in the study. They had abstained from smoking and drinking for 1 week prior to the test. All were aware of the experimental procedures and precautions before signing the informed consent form. The study was approved by the Bio- and Medical Ethics Committee of Dalian University of Technology (Approval Number: DUTSEPE220708_01). The room temperature of the experimental site was approximately 25°C, and the experimental period was chosen to be within 2 hours after 9:00 a.m. or 15:00 p.m.

### 2.2 Vibration experimental test bench

**FIGURE 1:**
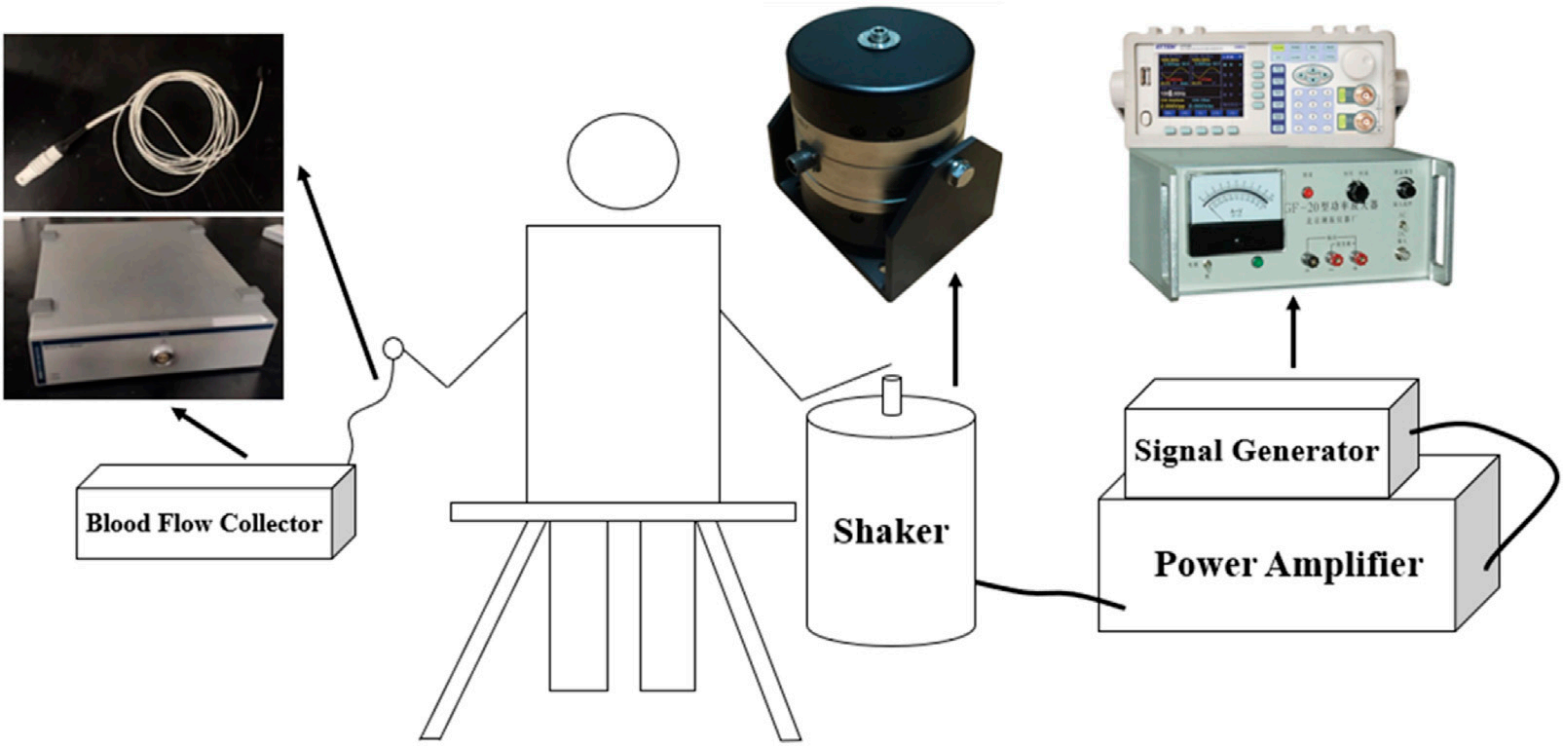
Hand-transmitted vibration test bench.

The vibration experimental test bench mainly consists of vibration device, including signal generator, power amplifier; shaker; blood flow acquisition device, including laser Doppler flowmetry (LDF), blood flow acquisition probe; handheld vibrometer; shaker connector; and PC. The signal generator and power amplifier were used to generate the vibration with given frequency and amplitude for this experiment. The LDF and blood flow acquisition probe were used to collect the fingertip skin blood flow data, and the handheld vibrometer was used to measure different vibration accelerations to evaluate the magnitude of vibration intensity. In particular, the columnar palm rest was printed by a 3D printer. The hand-transmitted vibration experimental platform is shown in Figure 1.

### 2.3 Experimental protocol design and experimental procedure

To measure the effect of the low frequency with varied amplitude vibration stimuli on fingertip blood flow in the vibrated finger and contralateral middle finger. The vibration frequency in the experiments were set to be 31.5 Hz, and the magnitude of vibration amplitude was evaluated by the root mean square of vibration acceleration according to the ISO 5349-2001 standard (ISO, 2001). In this study, four vibration acceleration stages were set to be 19, 36, 50, and 63 m/s^2^, and in each test there includes three time periods, namely a 5-min no-vibration period, a 5-min vibration period and a 15-min recovery period. During the experiments, after the vibration test in the previous stage of given amplitude value, the amplitude value of the next stage was adjusted. And the four stages were carried out in sequence with an interval of 25 min. The experimental protocol is shown in Table 1.

**TABLE 1:** Experimental protocol of vibration test.

Vibration frequency (Hz)	Unweighted acceleration magnitude (m/s^2^,rms)	Vibration condition		
		No vibration	Vibration	Recovery
31.5	19	0–5 min	5–10 min	10–25 min
	36	25–30 min	30–35 min	35–50 min
	50	50–55 min	55–60 min	60–75 min
	63	75–80 min	80–85 min	85–100 min

### 2.4 Processing and analysis of experimental data

In order to investigate the effect of vibration stimulation on the microcirculatory blood flow in the fingertip and the fluctuation of the main physiological regulatory frequency bands in the human body at different amplitudes, LDF-based fingertip blood perfusion level analysis and wavelet analysis were performed on the measured experimental data.

Blood perfusion levels of fingertip skin were first statistically analyzed within vibration stimulation. Wavelet analysis was used to quantify the changes of blood flow fluctuation amplitudes within three major regulatory frequency bands in the contralateral and vibrated hands at different time periods and amplitude magnitudes. Three regulatory frequency bands included endothelial regulation (0.0095–0.02 Hz), neural regulation (0.02–0.05 Hz) and myogenic regulation (0.05–0.15 Hz). The mean value was used as a measure of the centralized trend and the standard error was used as a measure of the dispersed trend, and the statistical methods were non-parametric and statistical significance analysis was performed using SPSS (*p* < 0.05 is considered statistically significant).

### 2.5 Analysis of endothelial cell response to vibration stimuli of different amplitudes

**FIGURE 2:**
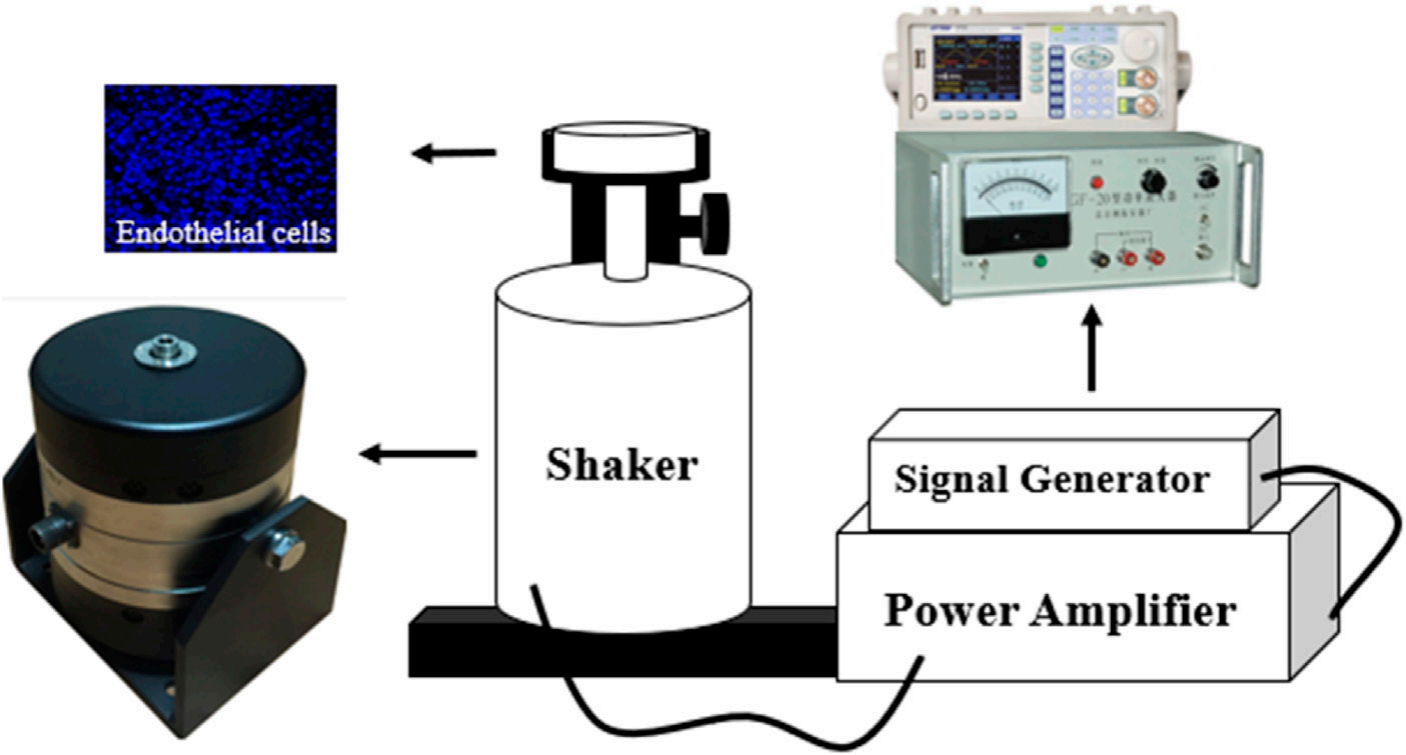
Endothelial cell vibration test bench.

In order to investigate the relationship between the vibration and the EC function, the EC morphology and its function was detected in the vibration stimulation experiments. The ECs were first cultured on the silicone film surface with ∼0.2 mm thickness in a culture dish in which the bottom center has a hole with a diameter of 20 mm cut off to expose the silicone film. The culture dish was then fixed to the shaker bolt with a bracket, and the vibration with different amplitudes acts on the silicone film. Three groups of culture dishes were assigned for the experiments, namely, the control group without vibration, vibration group I with 19 m/s^2^ and vibration group II with 63 m/s^2^, and the vibration duration was 20 min, the EC vibration test bench is shown in Figure 2.

## 3 Results

### 3.1 LDF-based analysis of fingertip blood perfusion levels

**FIGURE 3:**
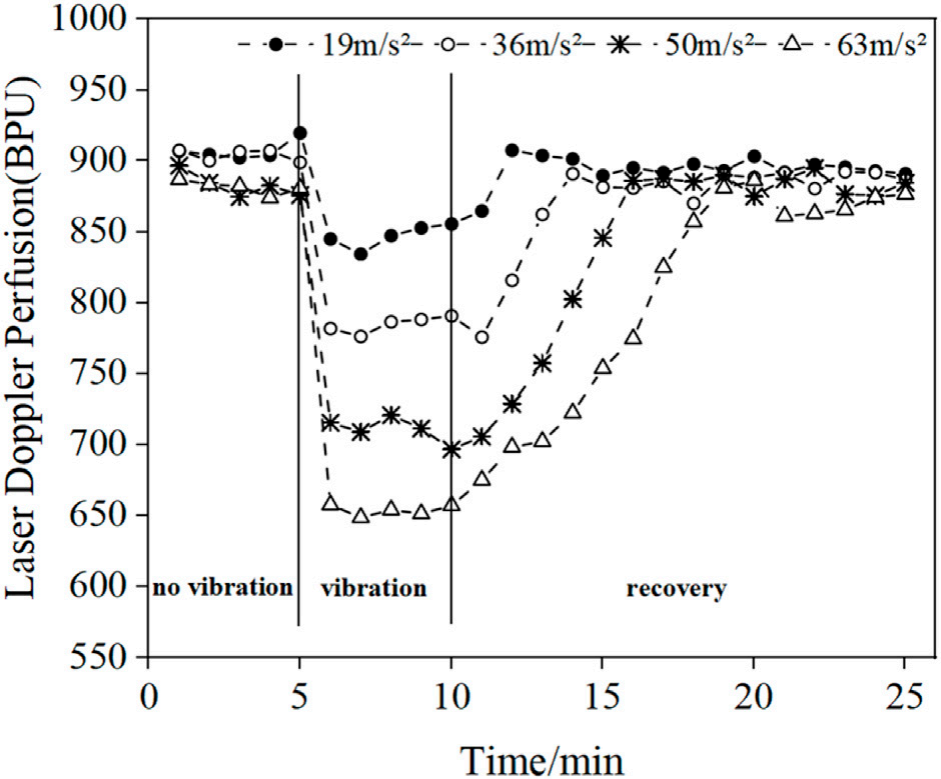
Changes in the mean blood flow values of the fingertips of the contralateral middle finger in eight subjects under different amplitude conditions.

Figure 3 shows the mean LDF blood flow values in the middle finger tip of the contralateral hand (left hand) of eight subjects. The mean blood flow value per minute was used to characterize the blood flow level during this minute, and the changes in blood flow before, during and after the vibration were observed.

The results in Figure 3 show that vibration stimulation will cause a decrease in blood perfusion level, and the blood perfusion level becomes lower and lower with the increasing amplitude of vibrations. In addition, after the vibration stimulation stops, the blood flow value will start to recovery the pre-vibration level, and the recovery time increases with the increase of amplitude.

**FIGURE 4:**
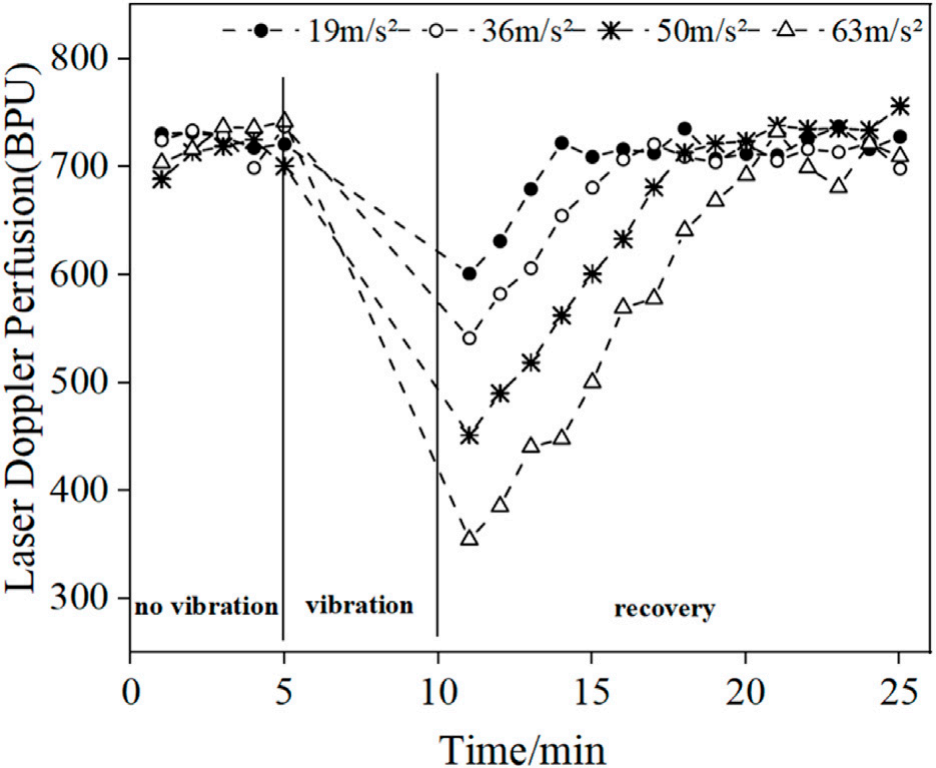
Changes in mean blood flow in the fingertips of the vibrated hand under different amplitude conditions.

Figure 4 shows the changes in the mean blood flow of LDF in the middle finger tip of the vibrated hand (right hand) in all subjects. Comparing the pre- and post-vibration blood flow values we found that the vibration stimulus caused a decrease in blood perfusion level, and as the vibration amplitude increased, the blood perfusion level decreased and the time required to return to the original state increased. Since the LDF blood flow acquisition probe was attached to the tip of the vibrated finger and would shake during the vibration period, resulting in errors in the blood flow signal, the data from the vibration period were excluded and only the pre- and post-vibration changes were compared.

**FIGURE 5:**
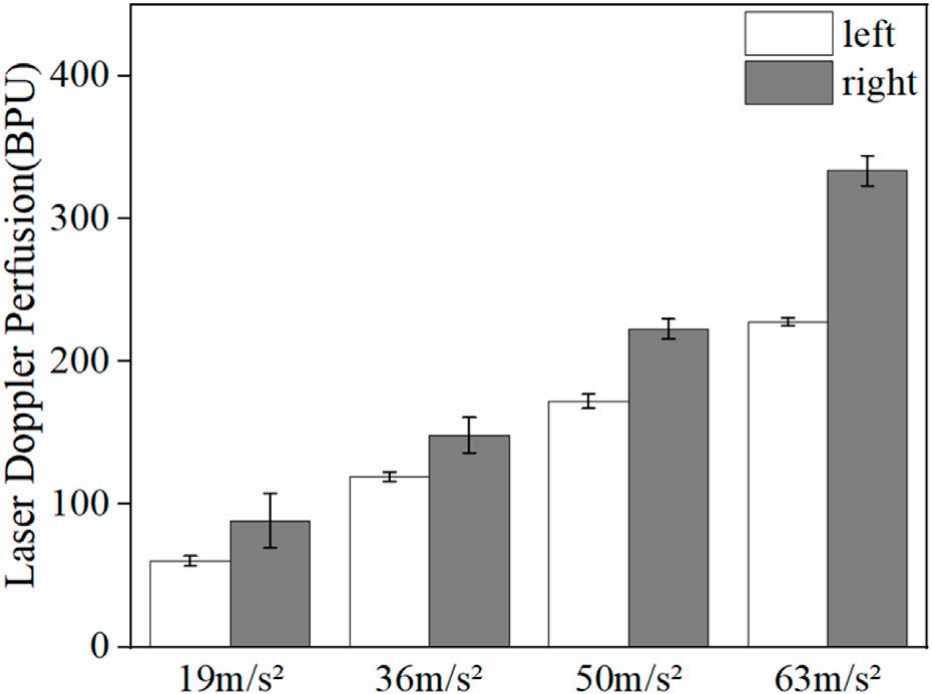
Comparison of reduced fingertip blood flow values in the contralateral hand and the vibrated hand.

Figure 5 shows the comparison of the reduction in mean LDF blood flow values in the middle finger tips of the contralateral and vibrated hands. The blood flow reduction is the difference between the blood flow value and the initial blood flow value during the vibration period and the vibration recovery period, respectively. The mean value of 5 min without vibration was taken as the initial blood flow value for the contralateral hand, and the mean value of 5 min during vibration was taken as the vibration period blood flow value, while the mean value of 5 min without vibration was taken as the initial blood flow value for the vibrated hand, and the mean value of 3 min after vibration was taken as the vibration blood flow value. It can be seen from the graph that the reduced blood flow value of the vibrated hand is greater than the reduced blood flow value of the contralateral hand, which can indicate that the vibration stimulation has a greater effect on the vibrated hand than the contralateral hand.

### 3.2 Fluctuation analysis of blood flow signal

It has been shown that endothelial band modulation, neural band modulation, and myogenic band modulation are three common physiological regulatory activities in the human body (Dunaev et al., 2014). In order to investigate the sensitivity of vibration stimulation on the regulation of each physiological frequency band in the human body, the fluctuation components of fingertip blood flow signals were extracted in the three main regulatory frequency bands of endothelial, neural, and myogenic in the contralateral hand and vibrated hand by wavelet analysis, and compared the fluctuation changes of each frequency band before, during, and after vibration.

**FIGURE 6:**
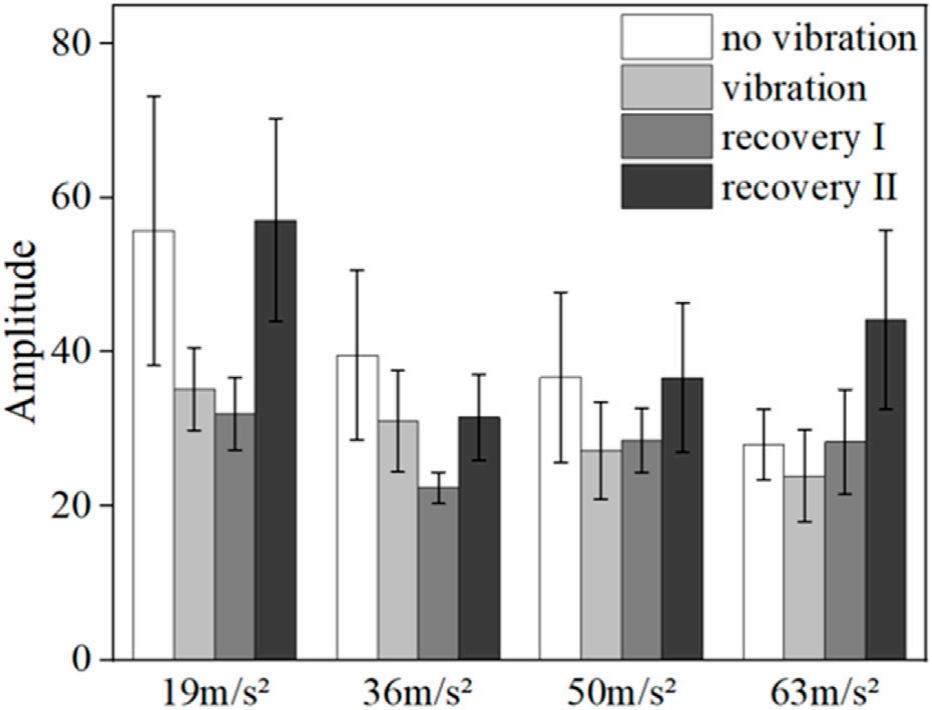
Mean blood flow fluctuation amplitude in the contralateral hand of all subjects at the neural frequency range.

**FIGURE 7:**
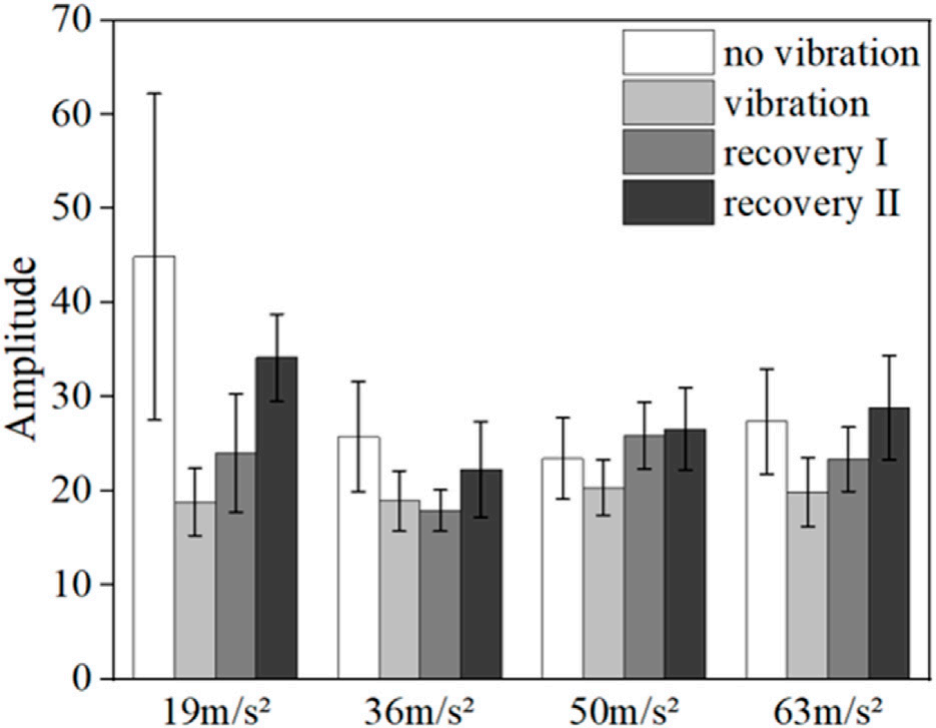
Mean blood flow fluctuation amplitude in the contralateral hand of all subjects at myogenic frequency range.

Figures 6, 7 show the mean blood flow fluctuation amplitude in the neuromodulation band and myogenic modulation band for the three phases of the contralateral hand for all subjects, respectively. The three phases are the no-vibration segment, vibration segment, and recovery segment, respectively. The recovery segment was divided into the post-vibration gradual recovery segment (Recovery segment I), and the post-vibration stable recovery segment (Recovery segment II).

From the results, it can be found that vibration leads to smaller fluctuations in blood flow in the neural (*p* = 0.032) and myogenic frequency bands (*p* = 0.037), but the pattern of changes in the amplitude of blood flow fluctuations at different amplitudes and in the amplitude of blood flow fluctuations in the recovery band is not obvious.

**FIGURE 8:**
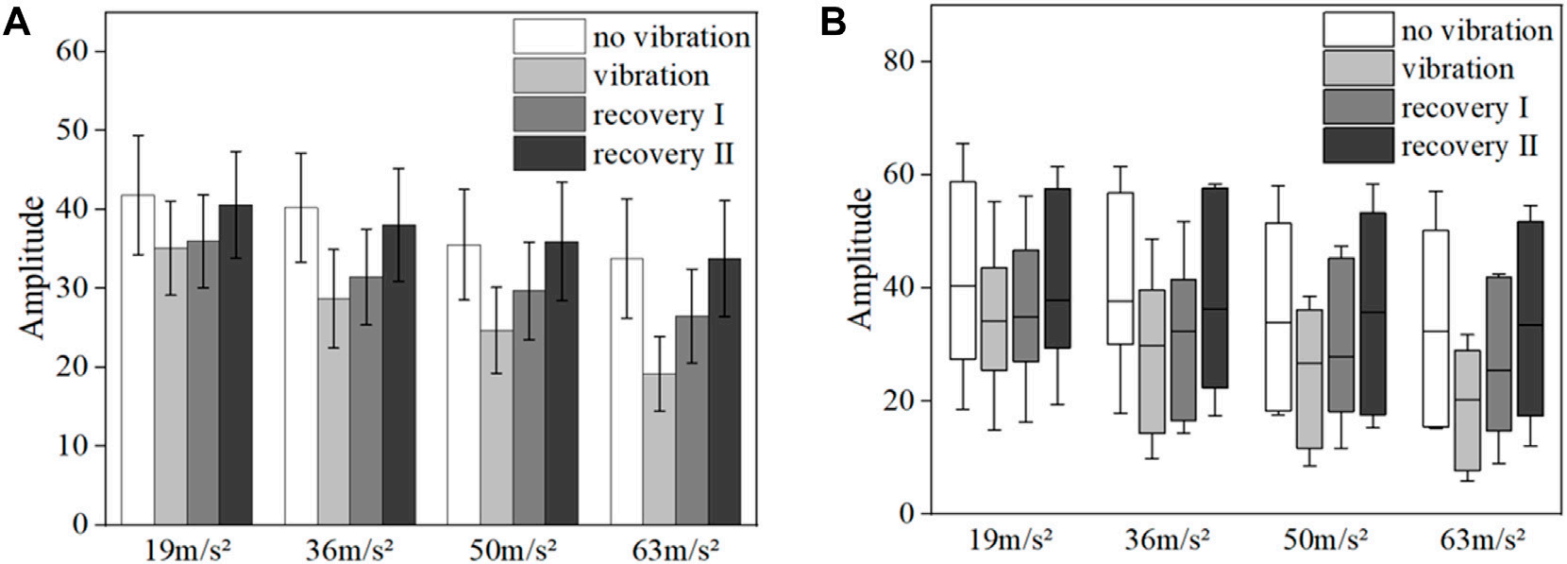
Mean blood flow fluctuation amplitude **(A)** and box line plot of blood flow fluctuation **(B)** in the contralateral hand of all subjects at endothelial frequency range.

Figure 8 shows the mean blood flow fluctuation amplitude in the endothelial frequency band of the fingertips of the contralateral middle finger of all subjects under vibration stimulation and the box line plot of blood flow fluctuation, respectively.

The results of the data in Figure 8A show that the fluctuation amplitude of blood flow tends to decrease under vibration stimulation regardless of the amplitude (*p* = 0.028). With the gradual increase of amplitude, the amplitude of blood flow fluctuation also gradually decreased (*p* = 0.024). The amplitude of blood flow fluctuation gradually increased in the post-vibration recovery section compared with that in the middle of the vibration (*p* = 0.031). In the post-vibration stabilization section, the fluctuation of blood flow was similar to the pre-vibration level. The box line plot in Figure 8B shows that the mean blood flow fluctuations in the endothelial modulation band decreased in the mid-vibration compared to the pre-vibration and post-vibration stabilization bands, and the median also decreased.

**FIGURE 9:**
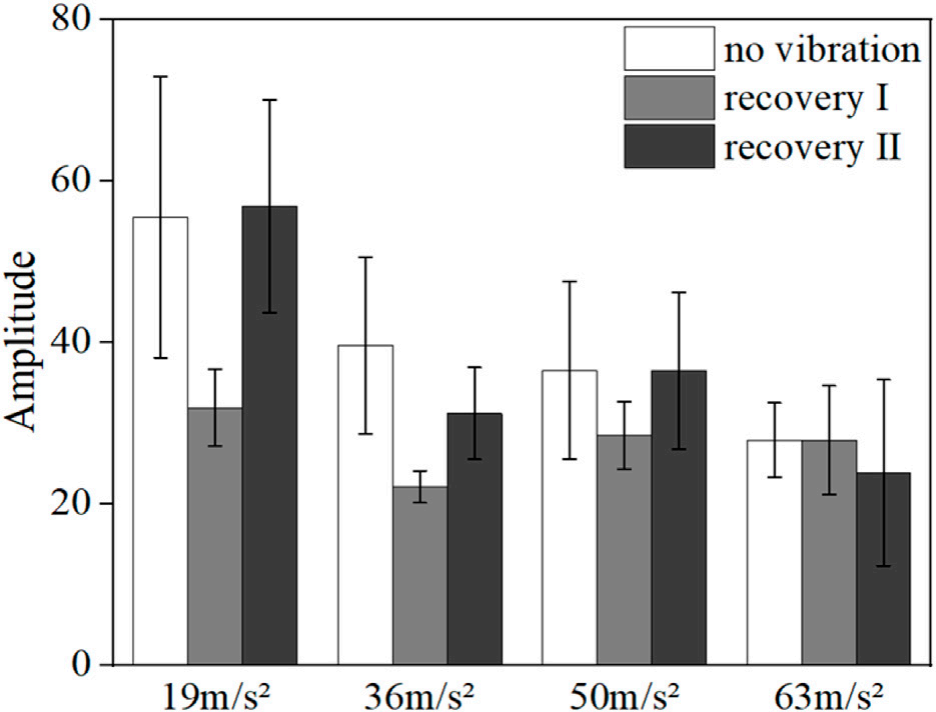
Mean blood flow fluctuation amplitude at the neural frequency range for the vibrated hand of all subjects.

**FIGURE 10:**
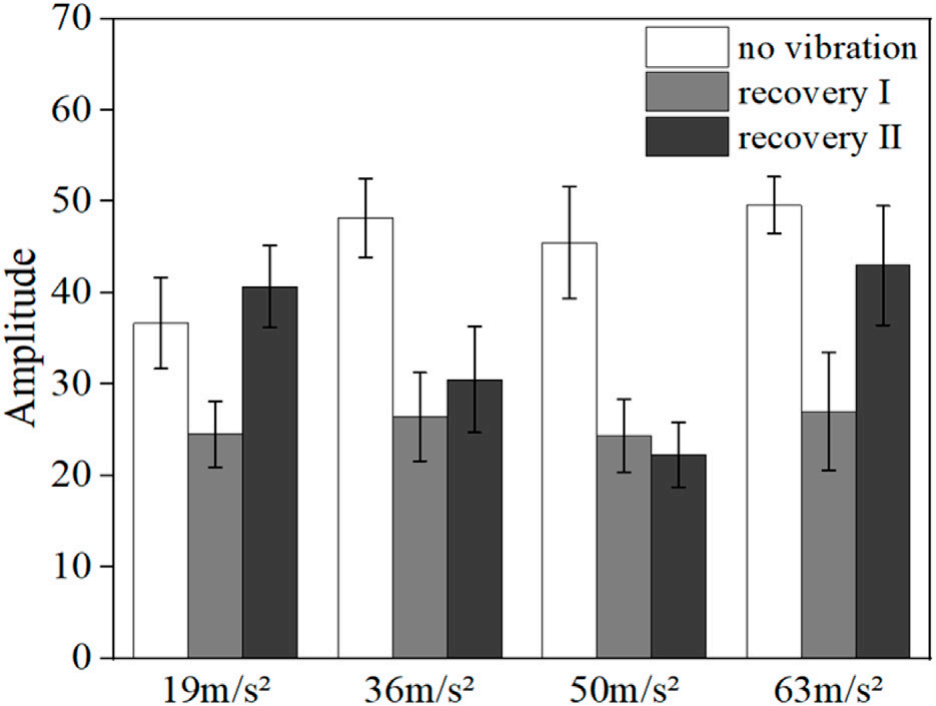
Mean amplitude of blood flow fluctuations in the myogenic frequency range in the vibrated hand of all subjects.

Figures 9, 10 show the mean blood flow fluctuation amplitudes in the neuromodulation and myogenic modulation frequency bands of the vibrated hand for all subjects, respectively. Under vibration stimulation, the blood flow fluctuation amplitude in the neuromodulation (*p* = 0.041) and myogenic modulation frequency bands (*p* = 0.029) showed a decreasing trend, and the effect of gradually increasing amplitude on the blood flow fluctuation amplitude was not obvious.

**FIGURE 11:**
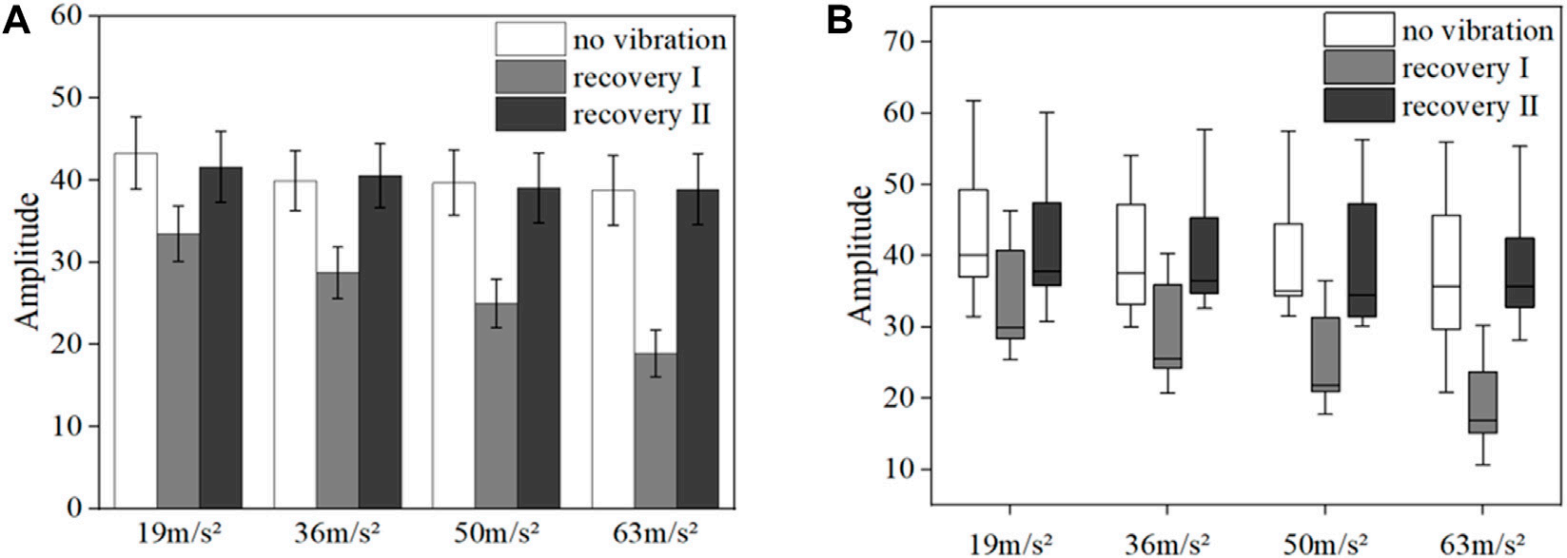
Mean blood flow fluctuation amplitude **(A)** and box line plot of blood flow fluctuation **(B)** in vibrated hand of all subjects at endothelial frequency range.

Figures 11A,B shows the mean blood flow fluctuation amplitude in the endothelial frequency band and the box line plot of blood flow fluctuation in the fingertip of the vibrated hand for all subjects under vibration stimulation, respectively. Among them, the results in Figure 11A show that the post-vibration recovery segment has less blood flow fluctuation than the pre-vibration segment (*p* = 0.017), and the blood flow fluctuation amplitude gradually decreases with the gradual increase of the amplitude (*p* = 0.021). In the post-oscillation stabilization section, the fluctuation of blood flow returns to a state similar to the pre-vibration level. The box line diagram shown in Figure 11B indicates that the blood flow fluctuations in the post-vibration recovery section of the endothelial frequency band decrease more significantly than those in the pre-vibration and post-vibration stabilization sections, and the median also decreases.

**FIGURE 12:**
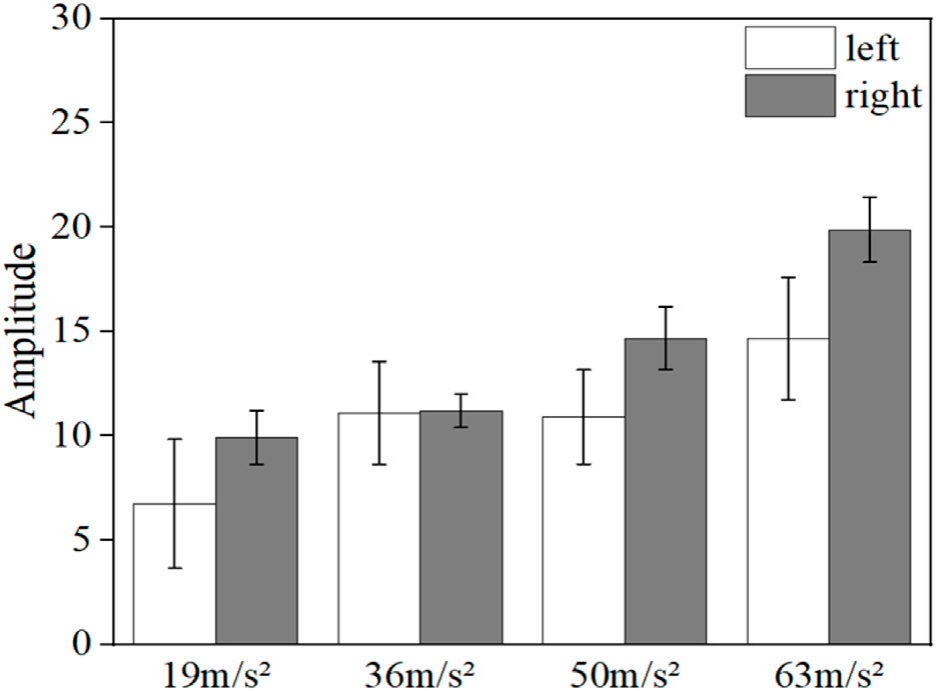
Comparison of the reduction in the amplitude of blood flow fluctuations in the contralateral hand and the vibrated hand.

Figure 12 shows the comparison of the mean blood flow fluctuation amplitude reduction in the middle finger tip of the contralateral hand and the vibrated hand. The blood flow fluctuation amplitude reduction is the difference between the initial fluctuation amplitude and the vibration fluctuation amplitude. The mean pre-vibration fluctuation amplitude was taken as the initial fluctuation amplitude for the contralateral hand, and the mean mid-vibration fluctuation amplitude was taken as the vibration fluctuation amplitude. And the vibration hand takes the average fluctuation amplitude before vibration as the initial fluctuation amplitude, take the average fluctuation amplitude of the recovery section after vibration as the vibration fluctuation amplitude.

It can be observed from the figure that the decrease in the fluctuating component of the vibrated hand was greater than that of the contralateral hand in all four conditions with different amplitude magnitudes (*p* < 0.05), indicating that the effect of the vibrating stimulus was more pronounced for the vibrated hand than for the contralateral hand.

### 3.3 Changes in endothelial cell morphology and inflammatory factor expression in response to vibration stimulation

**FIGURE 13:**
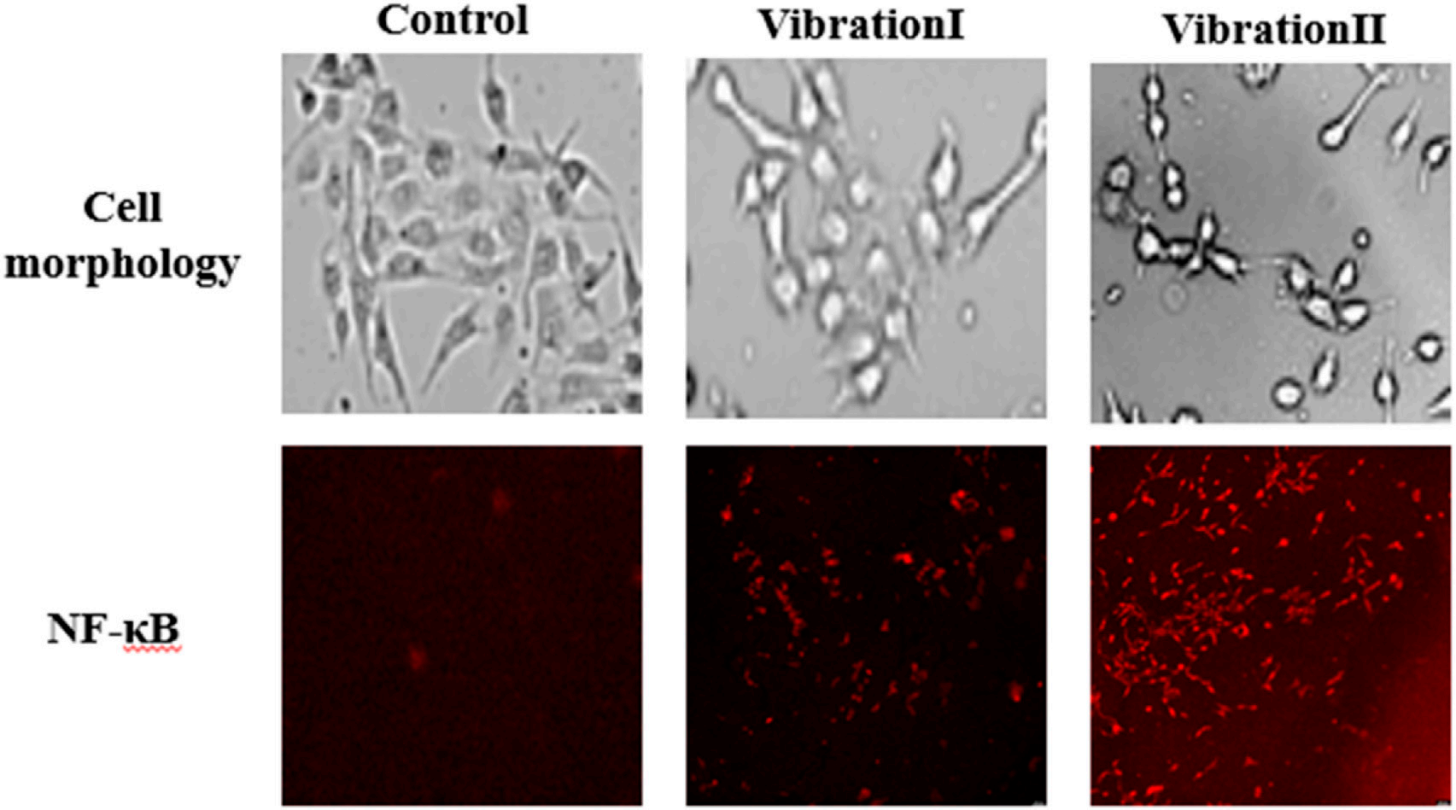
Cell morphology and expression levels of the inflammatory factor NF-κB.

The changes of endothelial cell morphology and inflammatory factor NF-κB under different amplitude conditions are shown in Figure 13. The results show that in the absence of vibration, the endothelial cells were flat, polygonal and closely arranged in a paving stone pattern. After the vibration stimulation, the cell morphology changed significantly. With the gradual increase of amplitude, the morphology of endothelial cells gradually changed to round, and the distribution density decreased, and the cells were irregularly arranged. In addition, compared with the control group without vibration, the vibration stimulation increased the expression level of NF-κB, and with the gradual increase of amplitude, the expression level of NF-κB also tended to increase gradually.

## 4 Discussion

The vibration experiment was performed in four amplitude phases, each of which included a 15-min post-vibration recovery period. From the results in Figures 2, 3, it can be seen that the blood flow in the fingertip skin recovered to a level similar to that before vibration within 15 min after vibration stimulation, which can indicate that the different amplitude stages used in this study are reasonable to be completed at once.

High-frequency low-amplitude vibration has a positive effect on tissue microcirculation. As Zhu et al. (Zhu et al., 2020) reported that vibration stimulation at a higher frequency (100 Hz) can significantly increase the level of blood perfusion in the toe, while vibration stimulation at a lower frequency (35 Hz) has no significant effect on the level of blood perfusion. This paper focus on the influence of vibration with different amplitude on blood flow. Therefore, a lower vibration frequency (31.5 Hz) is selected as the common vibration frequency of four different amplitude conditions.

The results of the LDF-based analysis of fingertip skin blood perfusion levels showed that the fingertip skin blood flow decreases under vibration stimulation and gradually decreases with the gradual increase of amplitude. At the end of vibration, the fingertip blood flow gradually returns to the pre-vibration level, and the length of recovery gradually increases with the gradual increase of amplitude. This is consistent with the experimental results of previous literature (Bovenzi et al., 1999). High-amplitude vibration causes vasoconstriction, resulting in increased blood viscosity and decreased blood flow (Xu et al., 2013). In addition, high-intensity vibrations also cause changes in human mechanoreceptors, such as pachytene vesicles (Thompson and Griffin, 2009).

The wavelet-based analysis of the skin blood flow signals of the middle finger tip at different vibration intensities showed that the vibration acceleration in the range of 19m/s^2^-63 m/s^2^ caused a significant decrease in the amplitude of the skin blood flow fluctuations in the endothelial modulation band. In addition, the amplitude of blood flow fluctuations in the endothelial modulation band gradually decreased with increasing amplitude, and the amplitude of fluctuations gradually returned to a level similar to the pre-vibration level at the end of the vibration. The change of blood flow fluctuation in the endothelial frequency band may be caused by the change of endothelial cells, which produce NO, which is rapidly transmitted to vascular smooth muscle cells through the cell membrane, causing smooth muscle cells to relax or contract, thus controlling the diastolic ability of blood vessels to change the fluctuation of blood flow (Tang and He, 2017). In contrast, under vibration stimulation, endothelial cells are damaged, leading to a decrease in NO concentration and consequent decrease in vasodilatory capacity and blood flow, so the average blood flow fluctuation in the endothelial band is weakened, and the greater the amplitude, the more serious the endothelial cell damage and the weaker the average blood flow fluctuation (Johnson et al., 2014).

Endothelial cell vibration experiments show that vibration stimulation causes significant changes in cell morphology and induces an inflammatory response in endothelial cells. The trend of both changes was more dramatic as the amplitude increased gradually. The changes in endothelial cell morphology and inflammatory factor expression levels lead to endothelial cell damage, which in turn leads to changes in endothelial regulatory activity and affects changes in blood perfusion levels in the microcirculation.

It has been reported that impaired biological activity or reduced synthesis of NO is the molecular basis of endothelial dysfunction, which can be used as biomarkers of vascular regulatory response (Harrison, 1997; Lespagnol et al., 2021). NO released by endothelial cells plays an important role in active relaxation of smooth muscle cells. The active regulation of blood flow could be realized by stimulating endothelial cells to secretory active substances affects smooth muscle cell tension (Furchgott and Zawadzki, 1980). Vibration induced endothelial dysfunction may lead to changes in NO concentration and then changes microcirculatory blood flow. It would provide an explanation for explaining why vibration stimulation changes microcirculatory blood perfusion.

This study has several limitations that should be noted. First, the subjects in this study were all male and the sample size was limited, and female subjects will be considered in later studies while continuing to expand the sample size. Second, the much longer recovery period would be needed. It can be found that after a recovery period of 15 min, the blood flow can be recovery to its original state. From the results it can be seen that the initial values of amplitudes are much lower than the initial one. And the LDF measures skin blood flow, and it is not known whether vibration affects deep tissue blood flow.

## 5 Conclusion

In this study, we analyzed the alteration of finger microcirculatory blood perfusion level by hand-transmitted vibration experiment combined with laser Doppler flowmetry (LDF) to measure the fingertip blood flow signals in the vibrated hand and the contralateral middle finger under the same frequency and different amplitude vibration stimulation, and examined the effect of vibration stimulation on the endothelial, neural and myogenic regulatory frequency bands in the fingertip based on wavelet analysis.

Analysis of blood perfusion levels showed that vibration stimulation causes a decrease in blood flow to the fingertip skin. As the amplitude gradually increases, the blood flow decreases. The length of time for blood flow to return to the initial condition after vibration increases accordingly. The results of wavelet analysis showed that the effect of vibration stimulation on endothelial regulation was more pronounced compared to neuromodulation and myogenic regulation. In the endothelial modulation frequency band, vibration stimulation causes a decrease in the amplitude of blood flow fluctuations in the fingertip skin, and the amplitude of blood flow fluctuations decreases gradually with increasing amplitude.

Finally, the results of the endothelial cell vibration experiment showed that the vibration stimulation had a certain effect on the morphology of the cells and the expression level of the inflammatory factor NF-κB, and the morphology of the cells changed more obviously with the gradual increase of the amplitude, and the expression level of NF-κB also increased.

## Data Availability

The original contributions presented in the study are included in the article/supplementary material, further inquiries can be directed to the corresponding authors.
